# Neonatal Hearing Rescreening in a Second-Level Hospital: Problems and Solutions

**DOI:** 10.3390/audiolres13040058

**Published:** 2023-08-15

**Authors:** Marta Gómez-Delgado, Jose Miguel Sequi-Sabater, Ana Marco-Sabater, Alberto Lora-Martin, Victor Aparisi-Climent, Jose Miguel Sequi-Canet

**Affiliations:** 1 Pediatric Department, Francesc de Borja University Hospital, 46702 Gandia, Spain; gomez_mardel@gva.es (M.G.-D.); marco_anasab@gva.es (A.M.-S.); albertoloramartin@gmail.com (A.L.-M.); victor.aparisic@gmail.com (V.A.-C.); 2 Rheumatology Department, La Ribera University Hospital, 46600 Alzira, Spain; sequi_jossab@gva.es; 3 Foundation for the Promotion of Health and Biomedical Research of the Valencian Community (FISABIO), 46020 Valencia, Spain

**Keywords:** hearing loss, neonatal hearing screening, rescreening, otoacoustic emissions, auditory potentials

## Abstract

Second-level hospitals face peculiarities that make it difficult to implement hearing rescreening protocols, which is also common in other settings. This study analyzes the hearing rescreening process in these kinds of hospitals. A total of 1130 individuals were included; in this cohort, 61.07% were hospital newborns who failed their first otoacoustic emission test after birth (n = 679) or were unable to perform the test (n = 11), and who were then referred to an outpatient clinic. The remaining 38.93% were individuals born in another hospital with their first test conducted in the outpatient clinic (n = 440). A high number of rescreenings were made outside of the recommended time frame, mainly in children referred from another hospital. There was a high lost-to-follow-up rate, especially regarding otolaryngologist referrals. Neonatal hearing screening at second-level hospitals is difficult because of staffing and time constraints. This results in turnaround times that are longer than recommended, interfering with the timely detection of hearing loss. This is particularly serious in outpatient children with impaired screening. Referral to out-of-town centers leads to unacceptable follow-up loss. Legislative support for all these rescreening issues is necessary. In this article, these findings are discussed and some solutions are proposed.

## 1. Introduction

In many countries, universal neonatal hearing screening is recommended or required by law. Since the birth of the Commission for the Early Detection of Hearing Loss (CODEPEH), a Spanish joint committee of pediatric and ENT experts, in 1995, various initiatives have been carried out with the aim of promoting and prioritizing the application of neonatal hearing screening programs throughout Spain. In 2003, a consensus was reached with the Ministry of Health and the Autonomous Communities to implement it, which has evolved through to the present day [1]. The Valencian Community has had a neonatal screening program since 2001 [2].

Newborn hearing screening protocols may vary by country and health system, but generally follow a similar structure. Usually, the program consists of screening all newborns before they leave the hospital or maternity center, although there are also out-of-hospital protocols. In many protocols, two-stage screening is applied. The first stage is a quick and simple screening test, such as the automated auditory brainstem response (AABR) or otoacoustic emission (OAE) test. If the baby does not pass the first stage, they will be referred for rescreening with these or other techniques a few days or weeks later (second stage). The rationale is that if a newborn fails that hearing screening test, it does not necessarily mean they have established hearing loss [3,4].

A retest before one month of life (confirmatory) is established in most neonatal hearing screening programs in order to reduce the number of infants referred for otorhinolaryngology (ENT) assessment (no more than 4%) [1] and to increase the specificity and sensitivity of the first step [3].

Failing in the first step test is highly variable and depends on several factors. There are many reasons why an infant may fail the initial screening test, such as amniotic fluid in the middle ear, a bad neonatal condition during the test, environmental noise, age at discharge, the member of staff that performs the test, and the technique used, among others [2].

Recommended follow-up after a failed hearing screening test is important as it can help to detect early hearing loss, ensuring that the child receives appropriate treatment and intervention services [5]. Children who do not receive timely intervention for hearing loss may experience delays in speech and language development, social and emotional development, and academic performance [2]. Detecting hearing loss early increases the likelihood of a successful intervention and optimal outcomes for the child. Depending on the severity and type of hearing loss, treatment options may include hearing aids, cochlear implants, and/or speech therapy [6].

Second-level hospitals face peculiarities that make the implementation of hearing screening protocols difficult, which are not uncommon in other settings, but may be aggravated by time and staffing constraints. In third-level hospitals, teams of several professionals involved in the screening process can be formed with sufficient time allocated. However, this is less frequent in second-level hospitals, causing worse performance and delays in recommended protocol, varying the final result. This can be particularly serious in the rescreening process.

Although the heterogeneity of the screening programs currently in place prevents generalizations based on one study, many of the problems encountered in a second-level hospital setting are indeed common to different hospitals and protocols. This study aims to analyze the hearing rescreening process from the point of view of quality criteria and objectives from programs generally accepted by specific committees, such as CODEPEH and JCIH (Joint Committee of Infant Hearing) [1,7].

This study aims to evaluate the following:

1. The rescreening volume and neonates’ origin;
2. The performance of rescreening in neonates born in the center’s maternity ward;
3. The performance of screening and rescreening in neonates born outside the center’s maternity ward;
4. To determine the rescreening times in both groups;
5. To know the final ENT diagnosis.

## 2. Materials and Methods

Inclusion criteria: All newborns born in this or other hospitals who underwent one or more neonatal hearing rescreening tests in the outpatient clinic of a second-level hospital from 1 January 2015 to 31 December 2022.

Exclusion criteria: Newborns who did not undergo any test in an outpatient clinic.

The study was approved by the hospital’s Ethics Committee with code e2/2018.

### 2.1. Protocol

The screening protocol discerned between neonates with risk factors and those without risk factors. The risk factor definitions provided by CODEPEH were used, based on Joint Committee on Infant Hearing (JCIH) recommendations [1,7].

In healthy newborns with no risk factors, the protocol was carried out in three steps. The first step consisted of a bilateral OAE test (first test) performing specifically transient evoked otoacoustic emissions (TEOAEs), at around 48 h of life, always within the abilities of the service. This was carried out every day of the week, depending on the service availability and the maternity ward nurses’ workload. Once oral parental consent had been obtained, the TEOAE test was carried out in the same room where the newborn lay, making sure that the environmental noise was as low as possible. In order to be sure that the newborn was calm, and therefore to avoid sedation in all cases, the test was performed after the newborn had been fed. In case of altered results, an outpatient appointment was made before one month of life to repeat the test (second test). If the results were normal, the baby was discharged, and if there was some alteration, an automatic auditory brainstem response (AABR) test was performed (third test). If those results were also altered, a referral was made to otorhinolaryngology (ENT) to carry out the diagnostic process and choose an appropriate treatment [2].

In neonates with risk factors, the first step was always the AABR (first test). In case of alteration, an outpatient appointment was made before one month of life to repeat the test (second test). If these also showed altered results, a referral was made to ENT for diagnosis and treatment, if needed [2].

Both techniques were bilaterally performed; if one of the ears was altered, it was considered as an altered test and, therefore, in the next test, both ears were retested. 

### 2.2. Techniques or Equipment

#### 2.2.1. Transient Evoked Otoacoustic Emissions (TEOAE)

From 2015 to 2018, TEOAEs were performed using an ECOCHECK OAE Screener^®^, and from January 2019 onwards they were collected using an OtoNova Screener^®^. Both are based on the ILO 88 system (Otodynamics Ltd. Hatfield AL10 8BB.UK) together with the ILO ECP^®^ neonatal probe. The technique consists of a 1ms stimulus whose intensity is 84 ± 3 decibels (dB spl) at a frequency of 80 cycles/s. An acceptable result required a signal greater than 6 dB above the ambient noise. The results are shown as either a pass or fail. Only bilateral passes were accepted as a normal result [2,8].

#### 2.2.2. Automatic Auditory Brainstem Response (AABR)

Until 2018, an AccuScreen^®^ MADSEN evoked potentials device (GN Otometrics, 2630 Taastrup, Denmark) was used for performing AABR tests. From 2019 onwards, the OtoNova Screener^®^ device was used. Three electrodes were placed (forehead, cheek and neck) and a series of stimuli was performed in each ear at an intensity of 35 dB HL. A threshold is the lowest level at which the wave V can be measured. The test results were considered impaired when this threshold was higher than 35dB HL. The results are shown as a pass or fail [9].

#### 2.2.3. Data Analysis

Data analysis was performed using an Excel 2010^®^ spreadsheet (Microsoft Co., Albuquerque, NM, USA) and the SPSS 20.0^®^ software platform (IBM Co., Armonk, NY, USA).

### 2.3. Study Variables

1. TEOAE test result in first and second test (normal/altered);
2. ABR test result (normal/altered);
3. Hospital of birth (internal/external);
4. Test in otorhinolaryngology (ENT) (normal/altered);
5. Intervals in days between the different phases of the screening.

## 3. Results

The distribution of cases is shown in Table 1 with total counts.

**Figure 1:**
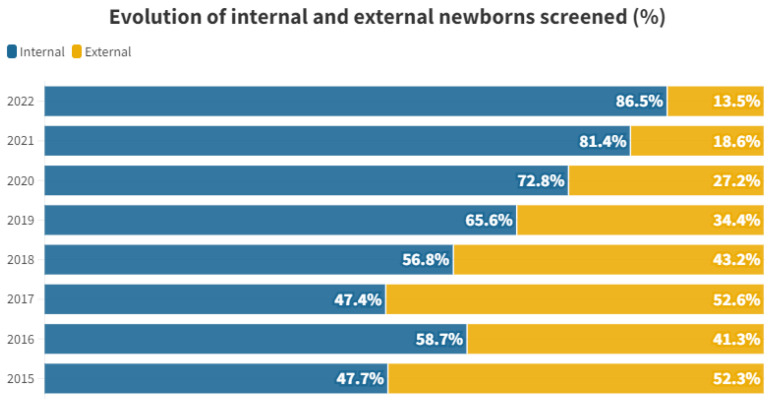
Percentage of internal and external newborns screened by year studied.

The proportional distribution of each year between internal and external individuals is shown in Figure 1.

Table 2 shows the mean time in days after birth for both groups and different tests.

**Figure 2:**
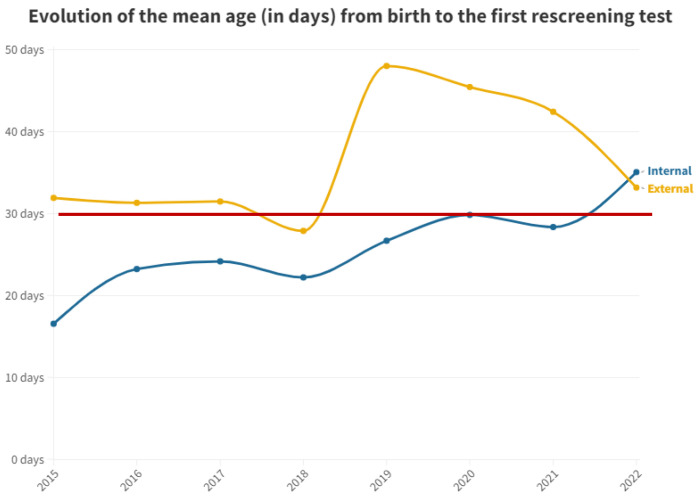
Mean number of days from birth to the first outpatient hearing loss test by year. The red line represents the desired limit for screening.

Figure 2 shows the mean time in days after birth until first outpatient rescreening test for both groups.

**Figure 3:**
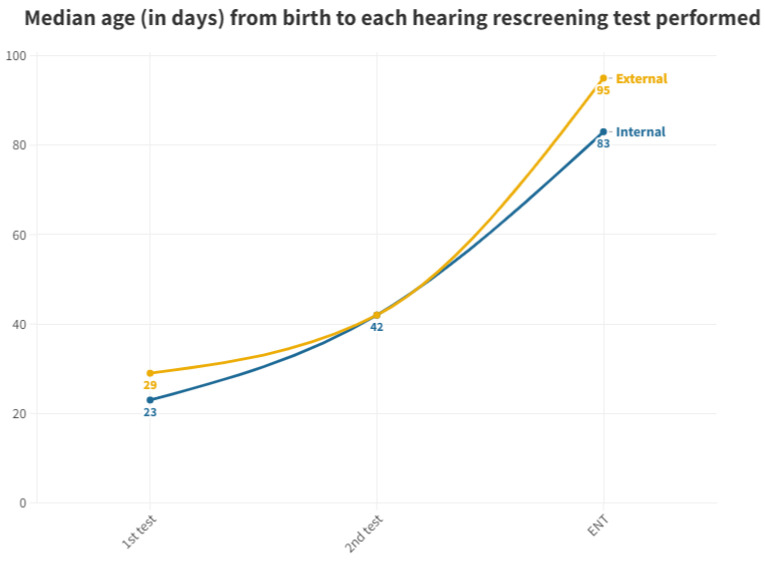
Median age at which each hearing screening test is performed in internal and external neonates.

Figure 3 displays the median, as it is a more representative value due to the presence of outliers in screened neonates who took a long time to be tested. 

Time elapsed between tests is shown in Table 3.

**Figure 4:**
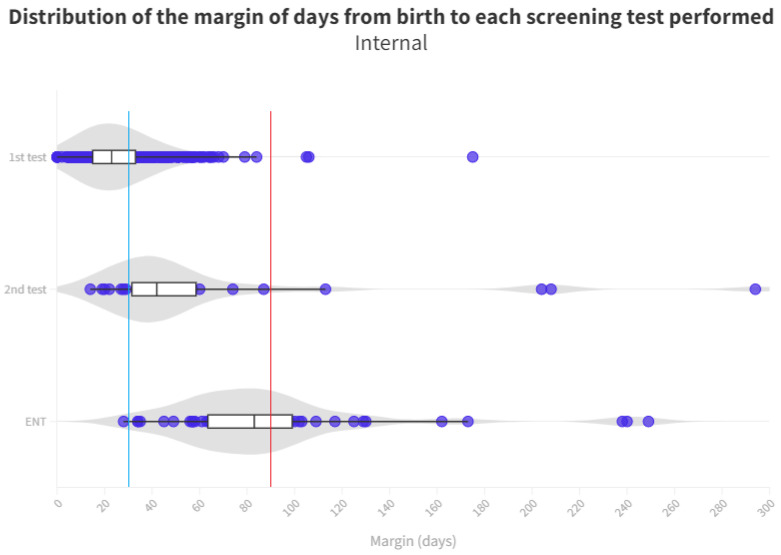
Distribution of the margin of days per test in internal neonates. The blue line represents the desired limit for screening. The red line represents the desired limit for ENT evaluation. Grey zones represent the distribution of cases.

**Figure 5:**
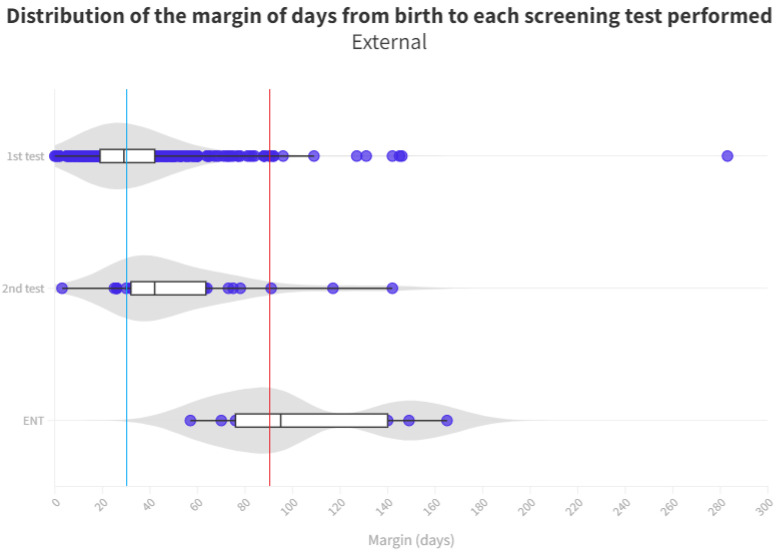
Distribution of the margin of days per test in external neonates. The blue line represents the desired limit for screening. The red line represents the desired limit for ENT evaluation. Grey zones represent the distribution of cases.

The time distribution for each test is shown in Figure 4 and Figure 5. Screening process limit (1 month) represents the blue line, whereas the red line is the ENT diagnosis limit (3 months).

Cases referred to ENT and their results are shown in Table 4.

Hearing loss cases after ENT evaluation are shown in Table 5.

Loss to follow up in both groups by phase is shown in Table 6.

## 4. Discussion

Neonatal hearing rescreening is a crucial part of the overall screening process that helps us enhance the outcomes and ensures precise detection of hearing loss in newborns. It provides opportunities for early intervention and assistance, but its development involves challenges, which we will discuss below.

### 4.1. Hospital of Birth of Screened Newborns

Hearing loss screening encompasses both newborns delivered at our hospital and those born at other institutions. From 2015 to 2022, a total of 8563 individuals were born at our hospital. Among these inpatients (“internal”), 690 (8.06%) were referred to the outpatient clinic for further hearing impairment screening. This referral occurred either because they did not pass their initial test in the maternity ward before discharge (n = 679) or because they were directly referred to the outpatient clinic (n = 11) without undergoing the initial test. Additionally, 440 individuals were born in other hospitals (“outpatients/external”) and were directly brought to our hospital’s outpatient clinic from primary care (see Table 1 and Figure 1). The study included a total of 1130 individuals.

When we examine the proportional distribution of internal and external individuals each year, we observe a certain consistency between 2015 and 2018. Starting from 2019, the proportion of tests conducted on internal individuals begins to rise, reaching 86.44% in 2022. This shift is attributed to a decline in the number of external newborns participating in the rescreening process. For external neonates, there is a peak in 2017, followed by a steep decline, with only 12 tests performed in 2022 (see Table 1 and Figure 1).

This trend is likely influenced by the enactment of decree 218/2018 on November 30th by the Regional Department of Universal and Public Health [10]. This decree governs screening programs within the Valencian Community, emphasizing that the initial hearing loss screening must be conducted at the birth hospital just before discharge. Private hospitals are also involved in administering this test, reducing the load on the rescreening process at public hospitals. While external screenings averaged at 47.35% during the 2015–2018 period, the percentage dropped to 23.42% between 2019 and 2022, showing a decreasing trend (see Figure 1). This underscores the significance of having legislation that supports comprehensive screening across society.

### 4.2. Rescreening Timing

During the process of rescreening, it is of utmost importance to manage the timing of each test to ensure effective program performance.

There exists a substantial disparity between the two groups in terms of the number of days between birth and the mean timing of the first test conducted, with distinctions seen in the inpatient and outpatient cohorts. While inpatients, following protocol, undergo testing shortly after birth (averaging 2.57 days), outpatients require approximately 33.91 days to complete their initial test at the outpatient clinic (see Table 2). In the case of inpatients who need to be rescreened (n = 679) or did not undergo testing in the maternity ward (n = 11), the time taken to complete the test at the outpatient clinic is 25.41 days. Consequently, inpatients can undergo two hearing screening tests before outpatients receive their first one.

To mitigate the impact of outliers caused by specific circumstances leading to abnormal test completion delays, the median was utilized as a test parameter.

As per the protocol, the initial screening test should be administered prior to discharge from the maternity ward. While 98.41% of neonates born at the hospital received their first test in the maternity ward, all neonates born at other centers and referred to the outpatient clinic from primary care miss out on this initial test before discharge. This discrepancy leads to a significant delay; while neonates born in the hospital have a median age of 2 days for their first otoacoustic emissions test in the neonatal ward, those born outside the hospital must wait for a median of 29 days to receive their first test, which is conducted directly in the outpatient department. The first outpatient test for those born in our hospital (due to abnormal results at maternity or those who were not tested) is conducted at a median age of 23 days. Thus, those born outside the hospital experience a 6 day longer wait for their first result (Figure 3).

For the second test carried out in outpatient clinics, the medians were equal at 42 days for both groups. This equality is attributed to the delay in the outpatient group, prompting pediatricians to expedite scheduling to confirm or negate altered results. Conversely, in the inpatient group, subsequent visits take longer, contributing to this observed equilibrium (Figure 3). It is pertinent to emphasize the increasing trend in the time elapsed before the first outpatient test for neonates born at this hospital. This trend has been consistent since data collection commenced in this study, indicating heightened pressure on medical services and reduced capacity to manage the workload, thereby emphasizing the need for additional staff dedicated to hearing screening.

Hence, it is imperative for healthcare institutions to prioritize neonatal hearing screening and allocate requisite resources to ensure prompt and effective screening. This might involve recruiting more personnel, delivering ongoing training and education to existing staff, and implementing initiatives for quality enhancement to streamline the screening process. Early detection and intervention of neonatal hearing loss can profoundly impact overall development and quality of life, making it a paramount concern for all healthcare providers [1,6].

With respect to neonates referred to Ear, Nose, and Throat (ENT) specialists, a notable disparity exists once again between those born in hospitals (median of 83 days) and those born outside (median of 95 days). During the analysis of ENT consultations, where patients who did not pass their tests receive a definitive diagnosis, a 12-day advantage is observed for those born in the hospital, allowing them to initiate treatment and adjustment sooner than their counterparts. The cause of this difference remains unexplained, considering both groups follow the same ENT referral procedure. It is worth noting that the third quartile for the outpatient group is 140 days, surpassing the 90 day threshold for ENT processes [11]. Consequently, while more than 50% adhere to the schedule, a significant number of infants do not receive their final results in a timely manner, with the third quartile being 99 days. As for those born outside the hospital, their median is already at 95 days, exceeding the aforementioned limit (Figure 5).

Timely and appropriate rescreening constitutes a critical aspect of neonatal hearing screening for the identification of hearing loss in newborns. Nonetheless, challenges can arise that hinder timely diagnosis and intervention.

Healthcare facilities may lack the staff or resources to perform diagnostic evaluations within the stipulated timeframe, leading to delays in diagnosis and intervention. Additionally, diagnostic hearing assessments can be intricate, necessitating specialized equipment and expertise. If the equipment is not properly calibrated or if the test is inadequately conducted, inaccurate results may ensue, necessitating subsequent repetition.

False positives occasionally arise, indicating hearing loss in a newborn when, in reality, there is none. Such occurrences can stem from a range of factors including ambient noise or the presence of debris in the ear canal. They can also result from unstable sleep patterns or excessive movement in newborns, making accurate testing impossible. These false positives introduce uncertainty and anxiety for families, along with the need for retesting. A high number of false positive results in newborns can increase strain on staff and lead to delays. In this context, the timing of the initial test is pivotal. The first few days of a newborn’s life are more prone to yielding a fail due to the significant impact of middle ear effusions on TEOAEs. In fact, certain studies [12] suggest that the pass rate for TEOAE, AABR, and combined TEOAE + AABR tests is highest in the 57–70 days and 71–84 days age groups, implying that delaying screening beyond 57 days may enhance re-screening pass rates and alleviate parental anxiety, which bears great clinical significance.

The issue lies in conducting the first test too late, which leads to a delay in the entire screening and diagnostic process, heightening the likelihood of missed follow-ups. Hence, administering the test during the initial days of a newborn’s life while still in the hospital is preferable.

A potential solution to truncate screening times might involve altering the protocol to incorporate a single initial AABR test before proceeding to the second level of referral. Discussions persist regarding the differences between one-step protocols employing AABR and two-step protocols involving TEOAE/AABR. When compared to TEOAE, AABR screening within 48 hours after birth can reduce the failure rate and false positive rate of the initial screening. However, when contrasted with two-step TEOAE screening, one-step AABR screening generates a higher rate of referral for audiological diagnosis, rendering it impractical, especially in hospitals with a high delivery rate. In such cases, the two-step TEOAE screening protocol remains applicable [13].

Equipment-related issues can also give rise to false positives. Regrettably, audiologists frequently take the various systems and their results for granted, despite significant disparities in performance across different systems. Numerous commercial TEOAE screening systems operate as “black boxes”, resulting in users having incomplete knowledge of their functioning. The stimulation parameters (stimulus shape, level, polarity), recording properties (windows, filters), and analysis method (classification criteria) often remain unspecified in user manuals. For instance, certain findings [14] reveal noteworthy disparities in TEOAE evaluation between the old and new versions of the Accuscreen device, even though they stem from the same manufacturer. Discrepancies were also evident when comparing Accuscreen devices with the ILO-292 system. This study underscores a disregarded issue pertaining to OAE recording systems. An unverified clinical consensus assumes that different OAE devices yield consistent outcomes, but the clinical reality differs considerably. Noteworthy differences exist between various OAE systems, even from the same manufacturer, highlighting the potential significance in generating false positive results.

Other factors contributing to such delays have been explored in other studies. Deng [15] identified a link between delayed hearing loss diagnosis and maternal education, maternal race/ethnicity, and neonatal intensive care unit (NICU) admission. Nikolopoulos [16] noted that delayed diagnosis and management, auditory neuropathy, late-onset deafness, and socioeconomic factors were major drawbacks of neonatal hearing screening programs. Chapman [17] found that the presence of co-occurring birth defects extended the duration of initial infant hearing screening, further exacerbating delays in subsequent hearing loss diagnoses.

### 4.3. Quality of Care

In a screening process where follow-up holds significant importance, the quality of care emerges as a pertinent indicator for evaluating the ease of family involvement. To gauge this, we have utilized the disparity in median days between consecutive tests, thereby negating the impact of exceptional cases and residual values. Smaller intervals signify higher quality of care.

As previously discussed, neonates born at this hospital undergo testing before those born externally, with the day of birth serving as the reference point. Those born in other hospitals experience a delay of 6 days before receiving their initial result (see Table 3).

In the transition from the first to the second test, inpatients exhibit a median interval of 19 days, signifying a substantial increase in time taken. Meanwhile, outpatients show a median interval of 13 days, possibly due to their delayed initiation of screening. In these cases, efforts are made to expedite scheduling for a new appointment upon detecting significant delays in the first test. Consequently, once outpatients become part of the screening process, they are scheduled for testing earlier than their hospital-born counterparts.

For ENT tests, the internal group undergoes the test 41 days after the last examination, while the external group experiences a delay of 53 days, either from the second test or if directly referred from the first (see Table 3). There is a median delay of 12 days in the final ENT diagnosis for neonates born outside hospitals compared to those born in our maternity ward (Figure 3). Thus, in the case of ENT diagnosis, internal neonates again require fewer median days than their external counterparts to reach this stage, receiving a definitive diagnosis and initiating adaptation in case of altered results. This disparity in days can significantly influence newborn development and the commencement of treatment. As previously mentioned, we lack an explanation for this.

Notably, the years of the COVID pandemic have not substantially altered the course of the screening protocol.

### 4.4. Screening and ENT Results

Through an examination of final ENT test results among neonates born at the hospital during the studied years, it was found that 58 individuals required an ENT appointment. These 58 patients constitute 8.41% of the rescreened newborns (n = 690) and 0.68% of the total births during the study period (n = 8563). Among these, 5 (8.62%) were lost to follow-up. Of the 53 with results, 64.15% (n = 34) received normal results, while 35.85% (n = 19) had altered results, a positive outcome for the screening process. Ultimately, within the hospital-born group, 97.21% of individuals achieved normal results, while 2.79% had altered ones over the entire period. This corresponds to a hearing loss incidence of 2.2‰ over the total hospital births during this timeframe, aligning with various studies [3,4] (refer to Table 4 and Table 5).

Among the 440 neonates born outside the hospital who underwent rescreening, only 9 required an ENT consultation, accounting for 2% of the total rescreened group. There were no missed appointments. In terms of test outcomes, seven of them (77.78%) obtained normal results, while only two (22.22%) were diagnosed with hearing loss, a noteworthy result in line with other studies [18] which found that ultimately only 24.4% of 545 infants had permanent hearing loss. Overall, for those not born at our hospital, 99.54% achieved normal results, with 0.46% yielding abnormal results. The patterns of normal and abnormal results seem consistent over time in both patient groups, although, in the case of outpatients, this becomes less relevant from 2018 onwards due to a reduction in individuals undergoing their initial screening in this hospital (refer to Table 4 and Table 5).

Regarding outpatient ENT consultations, where the final diagnosis is provided to patients, the contrast between the percentages of inpatient and outpatient referrals is notable. While 8.41% (n = 58) of hospital-born rescreened patients reach this stage, only 2% (n = 9) of outpatients do so. This disparity may stem from outpatients undergoing testing with more days of life, potentially leading to improved responses in the first test due to better middle ear conditions, resulting in fewer individuals requiring rescreening and referral to ENT (refer to Table 4 and Table 5). With these findings in mind, the proportion of neonates undergoing the confirmatory ENT test is not excessive, as the global rate should remain below 4%, whereas in our case it stands at 0.68% [11].

### 4.5. Losses (Lost to Follow Up)

In the context of hearing screening, “loss to follow-up” refers to a scenario where a neonate does not complete the recommended subsequent steps after the initial hearing screening test. Various studies [13] have highlighted that the proportion of infants failing the initial test and subsequently being lost to follow-up is a major challenge in neonatal hearing screening programs.

Rescreening infants who fail the initial test carries a risk of loss to the overall process, with reported rates ranging from 5% to 25%. When this figure surpasses 20%, it compromises the program’s validity [19,20].

A notable cause for loss to follow-up arises from birth in hospitals that do not conduct screenings. Consequently, these infants must be directed from primary care, potentially leading to non-participation in the screening process or, at best, exceeding the stipulated time limits for adequate screening (see Figure 4 and Figure 5).

According to our findings, the total losses amounted to 13 individuals (1.2%) out of the total 1130 patients studied (see Table 6). This figure falls within the range recommended by CODEPEH, which sets the maximum acceptable loss rate at 5% [19].

Examining losses in each specific test reveals an intriguing inverse trend between the two groups. At some point, 1.45% of inpatients discontinued the rescreening process. Notably, as the testing phases progress, the percentage of individuals lost to follow-up also increases. Neonates born in external hospitals exhibit a similar trend, albeit with a two-fold percentage of losses after the second test. Subsequently, they generally maintain attendance for future appointments. Overall, only 0.68% of outpatients abandon the screening process (see Table 5).

Among the 58 hospital-born neonates referred to ENT, there were five instances of loss, constituting 8.62% of the referred patients. This percentage is notably higher than the losses observed in the first test (0.58%) and the second test (1.54%). In contrast, among the nine patients born outside our hospital who were rescreened and referred to ENT, there were no instances of loss.

Certain studies propose several other factors contributing to loss of follow-up in neonatal hearing screening. For instance, Luz [21] identified forgetting the retest date and lack of awareness about the significance of retesting as notable reasons for non-compliance with newborn hearing screening retests. Davis [22] noted that inadequate information systems posed a major issue within the current UK practice.

Collectively, these studies underscore the necessity for improvements in information systems, tracking mechanisms, and public awareness to ensure effective program implementation and mitigate loss to follow-up in neonatal hearing screening. Some families might not fully comprehend the importance of these tests or the need for timely participation. They may also lack awareness about their child’s risk of hearing loss and the potential consequences of delaying diagnosis or neglecting testing and follow-up. Therefore, it is paramount for healthcare providers to educate families regarding the significance of follow-up appointments and vigilant monitoring for any potential signs of illness in their newborn, even if the initial screening yields normal results.

Furthermore, apart from awareness, challenges related to access to testing are evident. When a newborn fails the initial test, subsequent testing over the ensuing weeks is crucial to ascertain the extent and type of hearing loss, if any. However, some families may encounter difficulties in accessing these follow-up tests.

In the specific health area under analysis, barriers to access stem partially from transport to the hospital, especially for families residing in rural or remote areas or lacking reliable transportation. Our health area features a widely dispersed population, magnifying this issue [23].

Language barriers can also hinder families who do not speak the predominant language of the healthcare facility from understanding the screening process and comprehending the provided instructions. In the studied health department, 17.17% of the population comprises immigrants, a Figure 5.53% higher than the national average of 11.64% [24]. Additionally, the caregiver’s work–family balance plays a role, potentially complicating attendance at check-up appointments.

Various measures can address these barriers and ensure timely check-ups. Families should receive comprehensive information about the necessity of repeat testing when applicable and be available for addressing any queries they might have. The availability of translated materials or an interpreting service assists in effective communication with healthcare staff, ensuring accurate transmission of test-related information.

In terms of work–family reconciliation, flexible testing schedules should be provided, and transport systems or other resources should be accessible for families to attend appointments.

Collaboration with community organizations and outreach programs is crucial in identifying and addressing such impediments that obstruct proper neonatal screening.

If an infant becomes lost to follow-up in hearing screening, healthcare professionals should strive to engage with the family and encourage them to arrange a follow-up appointment. In certain cases, outreach programs or community resources can facilitate connecting families with the necessary services [25].

A notable occurrence is the observation of a significant hearing loss rate among school-age children, which is double the rate identified in newborns. Continuous awareness of late-onset hearing loss to enhance identification and hearing screening upon school entry is recommended. The potential implementation of universal hearing screening at different ages is being deliberated, and while strongly advocated, it could reclaim children who were lost to follow-up in neonatal hearing screening [26].

## 5. Limitations

This study did not take into account the hearing risk factors of the analyzed neonates. Irrespective of the protocol followed, the study aimed to assess the scope and effectiveness of neonatal hearing screening within a second-level hospital. Additionally, the difference between protocols lies in cases with risk factors, in which the initial test is conducted using AABR. If the results are abnormal in this initial test, a rescreening with AABR is also performed in outpatient clinics.

The outcomes acquired for TEOAE using the Echocheck Screener® and the OtoNova Screener®, as well as for ABR using the AccuScreen Madsen® and the OtoNova Screener®, indicate pass or fail results rather than actual values for response amplitude. However, even if this response is considerably lower, it might still possess adequate intensity for neonates to pass the test. For the TEOAE test results to be deemed impaired, a hearing loss greater than 30 dB HL is necessary, and in the case of ABR, a hearing loss greater than 35 dB HL.

The study presented herein reflects the practices within a specific hospital. Due to the diversity of screening protocols and programs across various hospitals and autonomous communities, it does not provide a comprehensive overview at a general level. Nevertheless, many of the issues discussed are relevant to different protocols, thus conferring its utility.

As the techniques utilized in hearing screening concentrate on a specific frequency range (0 to 6 kHz) at an intensity of 30–35 dB HL, further studies are needed to investigate other frequency ranges or intensities that may be affected but are not detected by these devices, hence not prompting rescreening referrals.

Although passing or failing the TEOAE test reflects the neonate’s hearing status, it is worth noting that, while the emitted signal is directly connected to the inner ear and cochlea, the middle and outer ear also contribute, as the signal traverses them. Consequently, disruptions in these areas could also manifest in the OAE test. Disparities in the number of false positive screening outcomes exist depending on the administered test [27], with ABRs demonstrating fewer false positives compared to TEOAE, the technique predominantly employed in this study.

The children referred from external hospitals may not all be eligible due to a lack of information on their overall numbers, the volume of tests conducted, or those lost to follow-up in the initial test.

In the current two-stage OAE/A-ABR newborn hearing screening protocol employed in this study, as with numerous other hospitals, the potential for false negative results exists. Certain studies conclude [28] that around 23% of those with hearing loss at approximately 9 months of age would have passed the AABR test. This discrepancy arises partly because much of the AABR screening equipment currently used is designed to identify infants with moderate or more pronounced hearing loss.

## 6. Conclusions

Conducting hearing screening within a second-level hospital is challenging due to staffing and time limitations characteristic of medium-sized hospitals. This results in turnaround times longer than recommended and obstructs timely hearing loss detection. Substantial administrative support is vital for the effective implementation of any screening program, considering its potential for cost savings.

Screening times differ among patients due to various factors, with one of the most significant being the neonate’s birth hospital. Delayed referral of outpatients from primary care for screening necessitates the performance of the first test and rescreening in their birth hospital or, alternatively, the establishment of an expedited pathway for outpatients from primary care or direct referral from the birth hospital to the designated hospital.

The rescreening process is delayed because appointments are typically scheduled around one month after birth (the time limit for the initial hearing screening). Unforeseen events and repetitions easily result in surpassing the recommended screening timeframe. If appointments were set within 15 days of birth for rescreening, there would be sufficient time for additional tests before the one-month limit, if needed.

Referring children with impaired screening outcomes to distant ENT referral centers leads to unacceptable loss to follow-up among these high-risk children.

Legislative support is essential to address all these rescreening challenges.

## Figures and Tables

**Table 1:** Evolution of neonatal hearing rescreening from 2015 to 2022, discriminating between internal and external neonates.

	2015	2016	2017	2018	2019	2020	2021	2022	Total
Internal	82	115	93	88	84	59	92	77	690 (61.07%)
External	90	81	103	67	44	22	21	12	440 (38.93%)
Total	172	196	196	155	128	81	113	89	1130

**Table 2:** Mean ($\bar{x}$) number of days elapsed from birth to the performance of each test in internal and external neonates.

	$\bar{x}$	
	Internal	External
Maternity	2.57	
1st test	25.41	33.91
2nd test	64.86	51.37
ENT	90.11	104.33

**Table 3:** Median times between tests in days.

Origin	Birth to 1st Outpatient Clinic Test	1st to 2nd Outpatient Clinic Test	to ENT
Internal	23	19	41
External	29	13	53

**Table 4:** Cases referred to ENT by group.

Origin	Normal		Altered	
Internal: n = 53 (85.48%)	34	64.15%	19	35.85%
External: n = 9 (14.52%)	7	77.78%	2	22.22%
Results	41	66.13%	21	33.87%

**Table 5:** Hearing loss after ENT evaluation (LFU not included).

**Results**	**Internal**	
Normal	661	97.21%
Altered	19	2.79%
	**External**	
Normal	435	99.54%
Altered	2	0.46%

**Table 6:** Losses in the screening (LFU) according to the test performed.

LFU	Cases	Losses	%
Internal	690	10	1.45%
1st test	690	4	0.58%
2nd test	65	1	1.54%
ENT	58	5	8.62%
External	440	3	0.68%
1st test	440	2	0.45%
2nd test	29	1	3.45%
ENT	9	0	0.00%
Total	1130	13	1.15%

## Data Availability

The data are not publicly available due to privacy and legal reasons.
